# Temporal lobe epilepsy associated with GAD autoimmunity

**DOI:** 10.1007/s00592-016-0910-9

**Published:** 2016-09-08

**Authors:** Atsushi Obata, Yumiko Kutoku, Yoshihide Sunada, Seizo Okauchi, Tomohiko Kimura, Hidenori Hirukawa, Akihito Tanabe, Tomoe Kinoshita, Kenji Kohara, Fuminori Tatsumi, Masashi Shimoda, Shinji Kamei, Shuhei Nakanishi, Tomoatsu Mune, Kohei Kaku, Hideaki Kaneto

**Affiliations:** 1 Department of Diabetes, Endocrinology and Metabolism, Kawasaki Medical School, 577 Matsushima, Kurashiki, 701-0192 Japan; 2 Department of Neurology, Kawasaki Medical School, 577 Matsushima, Kurashiki, 701-0192 Japan

Dear Editor,

Slowly progressive insulin-dependent diabetes mellitus (SPIDDM) and latent autoimmune diabetes in adults (LADA) are classified as a form of type 1 diabetes that has an autoimmune basis. They are also characterized by the presence of glutamic acid decarboxylase (GAD) antibody. GAD catalyzes the synthesis of gamma aminobutyric acid (GABA), which is the major inhibitory neurotransmitter in the central nervous system. Antibodies against GAD have been confirmed in patients with a number of neurological conditions such as stiff person syndrome, cerebellar ataxia, limbic encephalitis, myoclonus and epilepsy [1–4]. We herein report a case of a subject who had high titer GAD antibody and finally developed temporal lobe epilepsy. We also followed the change of GAD antibody titer for 3 years.

**Fig. 1:**
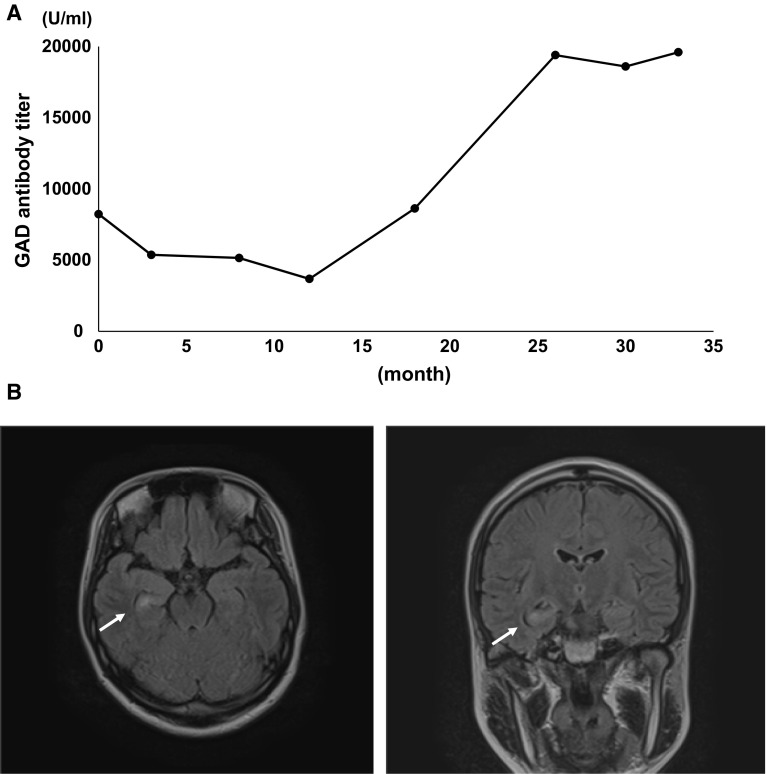
**a** Change of GAD antibody titer from the first visit to the onset of temporal epilepsy. **b** Brain MRI (FLAIR images) showing a high intensity area in right amygdala (*arrows*)

A 28-year-old female visited our hospital as her urinary glucose became positive during her pregnancy. She had no past medical history and no family history of epilepsy. On the first visit, she was in her second trimester pregnancy. In laboratory test, HbA1c was 5.5 % and fasting blood glucose level was 4.1 mmol/l. She was diagnosed with gestational diabetes mellitus after oral glucose tolerance test. In addition, serum GAD antibody was extremely elevated up to 8230 U/ml. These data suggested that she was complicated with SPIDDM/LADA. As her postprandial blood glucose level was high, we started insulin injection and she obtained good glycemic control and finally delivered a healthy baby. As glucose intolerance persisted even after the delivery, we continued insulin therapy. GAD antibody titer in this patient was constantly very high and was gradually further increased up to nearly 20,000 U/ml (Fig. 1a). About 3 years after the first visit when GAD antibody titer was nearly 20,000 U/ml, she felt tightness of the chest and palpitation which continued for a few minutes which was followed by the development of automatism of mouth and hands. She also had amnesia for automatism and conversation during the episodes. She underwent brain MRI, which revealed a high intensity area in right amygdala in FLAIR images (Fig. 1b). Although there were no obvious findings in electroencephalography, we diagnosed her with temporal lobe epilepsy from clinical symptoms and MRI findings. Paraneoplastic syndrome was ruled out by systemic imaging screening [5]. In laboratory examination, HSV, CMV, measles, mumps and EBV showed patterns of past infection and HIV was negative. Antinuclear antibody (ANA), anti-ss-DNA antibody, anti-ds-DNA antibody, anti-cardiolipin antibody, anti-SS-A antibody, anti-SS-B antibody, anti-cyclic citrullinated peptide antibody (anti-CCP antibody), proteinase3 anti-neutrophil cytoplasmic antibody (PR3-ANCA) and myeloperoxidase anti-neutrophil cytoplasmic antibody (MPO-ANCA) were all negative. Only anti-thyroglobulin antibody was positive (80.4 IU/ml). These results indicated no sign of active viral infection, other autoimmune diseases such as systemic lupus erythematosus, anti-phospholipid syndrome, Sjögren’s syndrome, rheumatoid arthritis and vasculitis. In cerebrospinal fluid (CSF) examination, CSF appearance was clear and cell counts, glucose concentration, total protein and IgG index were all within normal range: 2 cells/µl (100 % monocyte), 4 mmol/l, 0.25 g/l and 0.43 (normal range 0.3–0.7), respectively. PCR test for HSV, CMV, measles, mumps and HHV-6 in CSF was all negative. GAD antibody in CSF was elevated up to 425 U/ml. It is hard to rule out the other autoimmune antibody-associated encephalopathy as our screening did not cover all autoimmune antibodies. However, temporal lobe epilepsy associated with GAD antibody was strongly suspected in this case from its clinical course as temporal lobe epilepsy developed accompanied with further elevation of GAD antibody titer.

Taken together, we think that temporal lobe epilepsy was associated with GAD autoimmunity in this patient. After starting carbamazepine, her symptom was gradually mitigated. However, since it has been reported that GAD-associated central nerve system disorders are refractory and drug resistant, we think that we should be careful about the exacerbation of the disease in the future.

In conclusion, we propose that physicians need to pay attention to neurological symptoms in patients with high titer GAD antibody.
